# Fabry-Perot Pressure Sensors Based on Polycrystalline Diamond Membranes

**DOI:** 10.3390/ma14071780

**Published:** 2021-04-04

**Authors:** Sara Pettinato, Daniele Barettin, Vadim Sedov, Victor Ralchenko, Stefano Salvatori

**Affiliations:** 1 Engineering Faculty, Università Niccolò Cusano, Via don Gnocchi 3, 00166 Rome, Italy; sara.pettinato@unicusano.it (S.P.); daniele.barettin@unicusano.it (D.B.); 2 Istituto di Struttura della Materia, Consiglio Nazionale delle Ricerche (ISM-CNR), Sede Secondaria di Montelibretti, Via Salaria km 29,300, Monterotondo Stazione, 00015 Rome, Italy; 3 Prokhorov General Physics Institute, Russian Academy of Sciences, Vavilov Str. 38, 119991 Moscow, Russia; sedovvadim@yandex.ru (V.S.); vg_ralchenko@mail.ru (V.R.)

**Keywords:** CVD-diamond film, Fabry-Perot cavity, diamond-on-silicon diaphragm, pressure sensor, harsh environment

## Abstract

Pressure sensors based on diamond membranes were designed and tested for gas pressure measurement up to 6.8 MPa. The diamond film (2” diameter, 6 μm thickness)—grown by microwave plasma chemical vapor deposition on a silicon substrate—was a starting material to produce an array of membranes with different diameters in the 130–400 μm range, in order to optimize the sensor performance. Each 5 mm × 5 mm sensing element was obtained by subsequent silicon slicing. The fixed film thickness, full-scale pressure range, and sensor sensitivity were established by a proper design of the diameter of diamond membrane which represents the sensing element for differential pressure measurement. The pressure-induced deflection of the membrane was optically measured using a Fabry-Pérot interferometer formed by a single mode optical fiber front surface and the deflecting diamond film surface. The optical response of the system was numerically simulated using geometry and the elastic properties of the diamond diaphragm, and was compared with the experiments. Depending on the diamond membrane’s diameter, the fabricated sensors displayed a good modulation depth of response over different full-scale ranges, from 3 to 300 bar. In view of the excellent mechanical, thermal, and chemical properties of diamond, such pressure sensors could be useful for performance in a harsh environment.

## 1. Introduction

Static and dynamic pressure measurements are extremely important in many industrial areas, as well as in the biomedical, automotive, and aerospace fields. For example, pressure sensors used to measure reserves in oil reservoirs are currently being used to optimize production rates; pressure measurements made in vehicle engines are enabling pollution reduction and improved driving. In order to employ pressure sensors in different environments, several types have been developed: capacitive, piezoresistive, optical fiber grating sensor, piezoelectric, and others [1]. Currently, due to rapid technological evolution, all of those classes have good performance in terms of their high accuracy and linearity, good thermal stability, and a wide measuring range. Piezoresistive pressure sensors are now a mature technology, and they have a low manufacturing cost, high sensitivity, and good linearity characteristics [2]. The capacitive pressure sensors, although they have a high output impedance and a low anti-interference capacity [3], are widely used because their performance is temperature independent, and they have a low power consumption [4]. Fiber optic sensors are immune to electromagnetic interferences (EMIs) and are chemically inert; they also have good stability, even in relatively high temperature applications [5]. Piezoelectric pressure sensors are an interesting sensor class mainly in self-powered devices, as their operation does not require an external power supply [6]. A disadvantage is that they require complicated measurement setups and dedicated signal conditioning systems [7].

Furthermore, in many applications, such as power plants, gas turbines, and material processing systems, it is required to accurately measure pressure in-situ, even in hostile environments. Hostile environments mean a combination of high temperatures and the presence of aggressive media and corrosive gases. Traditional silicon-based pressure sensors do not perform well in hostile conditions; indeed, they have a limited maximum operating temperature around 482 °C, are sensitive to temperature variation, and are susceptible to EMIs [8]. Even silica-based fiber optic sensors developed for high-temperature applications, when used at temperatures above 800 °C, suffer severe performance degradation due to silica creep and dopant diffusion into the fiber [9]. Several solutions have been proposed to overcome the limitations of silicon-based technology using different materials and methods, such as diaphragm-ferrule-based optical fibers [8] or a sapphire-based pressure sensor. Sapphire, in particular, is considered a suitable material for high temperature applications due to its mechanical strength and chemical corrosion resistance, as well as having a very high melting point, i.e., 2040 °C [10,11]. In addition, silicon carbide (SiC) has been the focus of many works of research for several years for the realization of pressure sensors [12,13]), although its production costs are higher if compared to those of silicon technology.

In this context, diamond fits in as a particularly attractive material for pressure sensor realization to be used even in harsh and aggressive environments at relatively high temperatures, thanks to its particular physical, chemical, and mechanical characteristics. Diamond has many advantages over other electronic materials, being a wide bandgap semiconductor, i.e., 5.47 eV at room temperature. For this reason, it is particularly useful for the fabrication of devices which are able to operate in high temperature regimes [14]. Besides the ability to operate at high temperatures, it is chemically inert and radiation resistant due to its strong covalent bonding and the high displacement energy of atoms from its lattice (≈43 eV). Owing to its chemical-physical peculiarities, synthetic diamond produced by microwave plasma assisted chemical vapor deposition (MPCVD) techniques has received a great deal of attention in the design and implementation of radiation detectors. Indeed, polycrystalline and single-crystal CVD-diamond is currently used in the fabrication of X-ray [15,16,17], UV [18,19,20,21] and ionizing radiation [22,23,24,25,26,27,28,29] detectors. Moreover, diamond detectors have such distinctive characteristics that they are marketed as X-ray radiation dosimeters [30]. 

Modern techniques are used to grow both single crystal (SCD) and polycrystalline diamond (PCD) films, with characteristics which are very close to those of natural diamond [14,31]: high thermal conductivity (2200 Wm^−1^K^−1^), a low thermal expansion coefficient (1.0 ppm/°C), and a high value of the Young modulus (1143 GPa). The polycrystalline CVD-diamond is principally a gradient material: the diamond growth starts from small (often nanometer-scale) nucleation centers artificially deposited on the substrate; then, the primary crystallites evolve into vertically-oriented columnar grains, with the grain size increasing with the film thickness. The fine-grain layer on the substrate side is known to have an enhanced optical absorption [32] due to defects on grain boundaries, which have a relatively high surface area. Translucent (‘white’) PCD films with a thickness of less than 300 µm possess a high fracture strength—between 550 and ~2000 MPa (better than that of sapphire)—which increases with the decrease of the grain size (and, hence, with the decrease of the film thickness) [33]. This represents an important behavior for the fabrication of sensors based on thin membrane bending. In a vacuum, single-crystal diamond is stable when heating up to the temperature of 1700 °C, converting to graphite at higher temperatures. In contrast, PCD films show a progressive darkening at temperatures above 1300 °C as a result of the structural modification of the grain boundaries [34]. In the presence of oxygen, diamond undergoes etching due to oxidation; slight etching becomes noticeable at temperatures above 600 °C. This limits the diamond’s performance in air, at least in the case of long exposure. However, using oxidation-resistant protective coatings, such as oxide films [35], the operation temperature could be significantly increased. In the light of the above characteristics, CVD-diamond membranes are suitable for pressure sensor fabrication for use in aggressive environments [36,37].

Typically, for diamond-based pressure sensor realization, the piezoelectricity [38] and piezoresistivity [39] of boron-doped diamond have been exploited. In recent years, diamond sensors based on Fabry-Perot (FP) interferometry have also been proposed. These sensors have high sensitivity, chemical inertness, and are immune to EMIs [5]. Nanocrystalline diamond sheets have also been used as a protective layer for the optical sensor head, increasing the resistance to damage and allowing the measurement even in biological materials, in view of the biocompatibility of diamond [40]. A pressure sensor based on a polycrystalline diamond membrane has recently been proposed [41]. In this work, the systematic study of several sensors based on thin diamond diaphragms is presented. An optoelectronic system is employed in order to detect the displacement of the membrane subjected to a pressure difference on its two sides. The fiber coupling ensures complete immunity to EMIs. Due to the mechanical properties of diamond, and with the proper choice of the diameter and thickness of the diamond membrane, the realized sensors can detect very high pressures, up to tens of MPa. It should also be noted that the sensing element has only the diamond film exposed to the environment of which the pressure is to be measured. Therefore, the devices can find application in measuring high pressures in aggressive and high-temperature environments, such as those found, for example, in combustion engines. The following sections describe the performed work. In Section 2, the basic sensor structure and experimental set-up used for the characterization are described. The system requirements were evaluated, taking into account both the expected diamond membrane characteristics and the optical system adopted for the measurements. Analytical models and numerical simulations were used to predict the sensing performances in terms of membrane bending as a function of inlet pressure. Finally, the same section describes diamond film growth, membrane realization, and film characterization, as well as the procedure adopted for the sensor fabrication. According to the design requirements, a set of six diamond-diaphragms was selected in order to illustrate the sensing performance in Section 3. The experimental results were compared with numerical simulations allowing the evaluation of the elastic properties of diamond membranes. The conclusions are reported in Section 4.

## 2. Materials and Methods

In this section, the design choices and material characteristics are illustrated for the realization of optical fiber coupled pressure sensors based on thin polycrystalline diamond membranes. Starting from the sensor’s specifications, the procedures and methods used for the sensor fabrication are described in detail.

### 2.1. Sensor Structure and Preliminary Specifications

**Figure 1:**
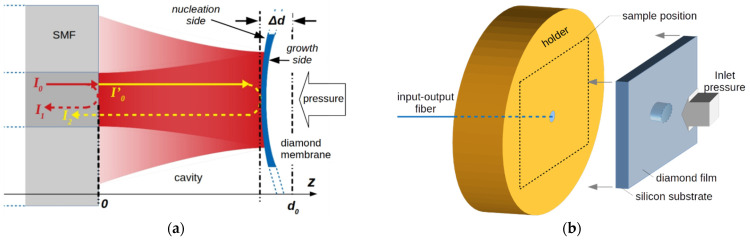
(**a**) Schematic of the FP-cavity of the sensor. The interference signal is generated by reflected beams at the fiber end (*I*_1_) and the diamond membrane (*I*_2_). (**b**) A sketch of the sensing element structure. A thin diamond membrane on silicon is aligned to a single mode fiber to realize a low-finesse FP cavity. The cavity length is equal to the silicon substrate’s thickness.

Figure 1a illustrates the basic principles of the pressure sensor used in this work. The structure exploits the feasibility of polycrystalline diamond film growth on silicon substrates, followed by the substrate’s selective area removal to produce a membrane. A thin diamond diaphragm on axis with a fiber termination is used to detect the magnitude of the pressure difference Δ*P* that can deform the diaphragm. A single-mode fiber (SMF), used as the input–output fiber, and the diamond diaphragm, used as a reflector, form an air gap which acts as a low-finesse FP cavity. The cavity has a length *d*_0_ when the diamond diaphragm is not deformed by pressure, whereas it is reduced by Δ*d* as a function of Δ*P*. Impinging light, with optical intensity *I*_0_—defined as the optical power per unit area—travels through the fiber. A portion of *I*_0_ is reflected off the fiber/air interface (*I*_1_), whereas the remaining light propagates through the air gap between the fiber and the diamond diaphragm surface, and is reflected back into the fiber (*I*_2_). The constructive or destructive interference of the two *I*_1_ and *I*_2_ light waves in the FP-cavity depends on the path length difference traversed by each wave, which is a function of the pressure difference on the diamond membranes which are able to reduce the FP-cavity length. The resulting light signal then travels back through the same SMF to a detector, where the signal is demodulated to produce a photocurrent amplitude as a function of the inlet pressure. In Figure 1b, a schematic view of the adopted structure is shown. Thin diamond film grown on a silicon substrate realizes a freestanding membrane after the selective etching of a hole in the substrate. The diamond-on-silicon membrane plate is then fixed to a sample holder where the SMF is inserted. The membrane geometry is then defined by the diamond film thickness and silicon hole diameter, whereas the silicon substrate thickness defines the FP-cavity length *d*_0_ when the inlet pressure is zero.

Optical interference would be mainly induced by the center deflection of the membrane if the light spot is perfectly aligned to the membrane axis, and if its diameter is negligible in comparison to that of the diaphragm. In such a case, the maximum sensitivity is gained by the sensing element.

**Figure 2:**
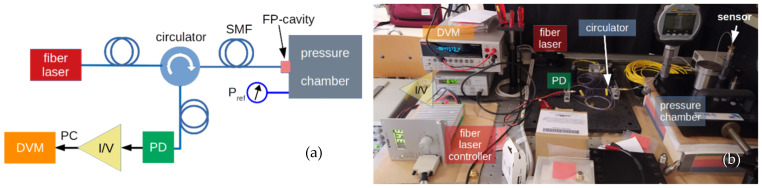
(**a**) Schematic (adapted from ref [41]) and (**b**) picture of the experimental set-up for the pressure sensor characterizations. An IR fiber-laser is coupled to the FP-cavity by means of a circulator. The interference signal is detected by a photodiode (PD). The photocurrent (PC) signal is acquired as a function of the pressure by a computer-controlled digital voltmeter (DVM) connected to the output of a current-to-voltage (I/V) amplifier.

Figure 2 illustrates the schematic and a photo of the set-up adopted for the sensor characterization. A fiber-coupled laser diode supplies coherent light to the sensing element via an optical circulator. The reflected light is then detected at the second leg of the optical circulator by the photodiode, and the photocurrent signal is converted into a voltage by the I/V amplifier. A computer-controlled digital voltmeter is finally used for the signal acquisition and processing. The experimental set-up includes the choice of light source and the type of fiber to be used. The most common type of SMF used for telecommunication has a core diameter of 8–10 µm, and is designed for use in the near-infrared (1.31 µm and 1.55 µm). In this case, the fiber core is so small that only light rays around 0° of incident angle pass through the fiber without much loss. Then, this type of fiber satisfies the requirement of collecting the light wave orthogonally reflected by the diamond membrane, giving a clearer inference signal and allowing SMF-membrane alignment, as described in Section 2.3. Modern SMFs have optimized the 1550 nm window, although dispersion is low at 1310 nm, too. For our purpose, a 1.55 µm SMF-pigtailed laser diode (LPSC-1550-FC, Thorlabs Inc., Newton, NJ, USA) was chosen as the light source, whereas an InGaAs infrared photodiode (IR-PD) (PD, FGA01FC, Thorlabs Inc., Newton, NJ, USA) was mounted to generate the photocurrent signal proportional to the light intensity of the two interfering light waves. A three-ports optical circulator (6015-3, Thorlabs Inc., Newton, NJ, USA) completes the optoelectronic section of the system.

Without any loss of generality on the sensor’s basic principle, the photocurrent signal generated by the PD can be estimated whilst neglecting the attenuation introduced by the optical circulator. Assuming the FP-cavity as a two-wave interferometer, hence neglecting the contribution of reflected light at the diamond film growth side (see Figure 1a), the intensity of light detected by the IR-PD is given by [42] (pp. 64–65)

(1) $$I=I_{1}+I_{2}+2\sqrt{I_{1}I_{2}}\cos\left(\Delta \varphi \right)$$

where Δφ is the phase difference between the two reflected waves. Hence, the output signal has a sinusoidal behavior, and its peak-to-peak amplitude—named the modulation depth—and the offset depend on the relative *I*_1_ and *I*_2_ intensities. As indicated in Figure 1a, if the Δ*d* expresses FP-cavity length-decrease due to diaphragm deformation, Equation (1) can be rewritten as

(2) $$I=I_{1}+I_{2}+2\sqrt{I_{1}I_{2}}\cos\left(\varphi_{0}+2\pi \frac{2\Delta d}{\lambda }\right)$$

where λ is the wavelength of light and φ_0_ is the phase shift between the two light waves for Δ*d* = 0, i.e., for a diamond diaphragm which is not exposed to a differential pressure. For the source wavelength of 1.55 µm, the fringe period, i.e., a phase change of 360 degrees, is equal to 775 nm. Then, by tracking the output signal, very small diaphragm displacements can be determined. The main drawbacks of this measurement system are the non-linear transfer function and the directional ambiguity of the output signal due to the sinusoidal behavior of Equation (2). The approach taken here to overcome the mentioned problems is to design the sensor so that, at the maximum membrane displacement, the signal does not exceed the linear region of the transfer function, or, at least, the range in which there is monotonicity, i.e., Δ*d* ≤ λ/4. It should be noted that more complex systems, such as dual-wavelength or broadband interferometry, could also be adopted [43]. In Equation (2), the modulation depth of the output signal depends on the geometric mean of light intensities, and is equal to 4(*I*_1_*I*_2_)^1/2^, whereas the offset value is equal to *I*_1_ + *I*_2_ − 2(*I*_1_*I*_2_)^1/2^. The maximum for the modulation depth is found for *I*_1_ = *I*_2_, where the offset nulls. Then, *I*_2_/*I*_1_ represents a figure of merit of the sensing element, with the best condition being found for a ratio equal to 1. Such a condition can be satisfied by the proper design of the cavity length, taking into account the reflectance values of the materials, as discussed below.

Affecting both the sensitivity and the dynamics of the sensor, the reflection and transmission at FP-cavity interfaces, as well as the light-beam spread at the SMF output, will be taken into account for the proper device design. For a rough estimate of the FP-cavity length, let us assume for the glass fiber a reflection of around 4% (refractive index *n* = 1.5). Conversely, diamond shows a refractive index of 2.38 in the near-infrared region [44], resulting in a reflectance of around 17%, although values up to 30% are found for polished polycrystalline CVD-diamond film [45]. However, the non-negligible spread of the fraction of light, which exits from the fiber, determines most of the light loss in the cavity. Indeed, assuming a Gaussian profile for the light beam, the dependence of the beam radius on *z* is given by [42] (pp. 83–84)

(3) $$w\left(z\right)=w_{0}\sqrt{1+{\left(\frac{z}{z_{0}}\right)}^{2}}$$

with its minimum value *w*_0_, which represents the beam waist, in the plane *z* = 0 (SMF end face in Figure 1a), and with *z*_0_ of the Rayleigh-range expressed as

(4) $$z_{0}=\frac{\pi w_{0}^{2}}{\lambda }$$

where the beam area is doubled. Then, 2*w*_0_ is the spot size of the output beam (*I’*_0_) which can be assumed to be equal to the diameter of the SMF core. For *z* >> *z*_0_, *w(z)* is almost proportional to *z*, as found by a more conservative approach considering the numeric aperture (N.A.) of the fiber, which simply gives *w(z)* = *z*·N.A. For SMF with a core of about 10 µm in diameter, specifically designed for λ = 1.55 µm, N.A. has a typical value in the range 0.11–0.14. For a cavity length of 100 µm, for example, a beam radius of 11 µm is calculated by Equations (3) and (4), in excellent agreement with the value estimated with an N.A. = 0.11. It is worth noting that, as a rule of thumb, the spot size of the beam reflected by the diamond membrane and impinging on the SMF would be of the order of 22% of the FP-cavity length.

If *P*_0_ is the optical power of the light at the fiber–cavity interface, the light intensity of the beam transmitted into the cavity is governed by

(5) I(z)=P0(1−RF)πw02[1+(zz0)2]

where *R_F_* is the reflectance of the fiber. Then, with 2*d* being the path of light reflected from the diamond membrane towards the SMF–input face, we obtain

(6) I2=P0(1−RF)2RDπw02[1+(2dz0)2]

whereas

(7) $$I_{1}=\frac{P_{0}R_{F}}{\pi w_{0}^{2}}$$

Hence, the *I*_2_/*I*_1_ ratio is expressed as

(8) $$\frac{I_{2}}{I_{1}}={\left(1-R_{F}\right)}^{2}\frac{R_{D}}{R_{F}}{\left(1+\frac{4d^{2}}{z_{0}^{2}}\right)}^{-1}$$

According to the previous equation, the FP-cavity length should be in the 43 and 62 µm range (*R_D_* equal to 17% and 30%, respectively) for *I*_2_/*I*_1_ = 1, assuming for *w*_0_ the SMF core radius. However, such low values for silicon thickness do not assure substrate planarity. A planar diamond-on-silicon plate is necessary for sensor assembly, as well as SMF-diaphragm alignment, as described in Section 2.3.

### 2.2. Diamond Membrane Design and Fabrication

**Figure 3:**
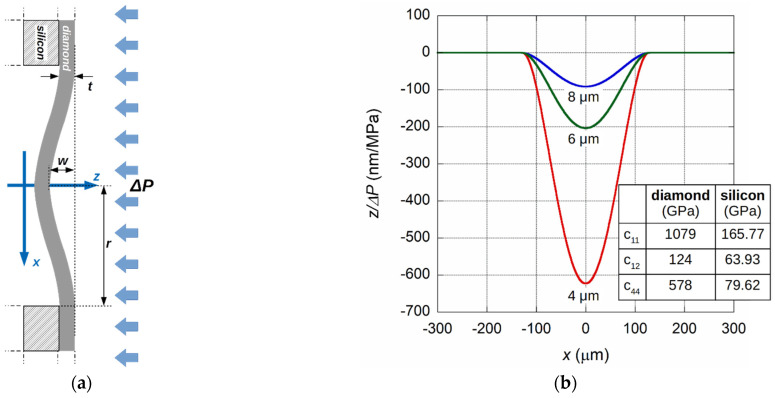
(**a**) Schematic of suspended diamond on a silicon diaphragm under the applied differential pressure. (**b**) Numerical calculation of the diamond membrane deflection normalized to inlet pressure for 4, 6, and 8 µm thick membranes with a diameter of 250 µm. Inset: elastic constants *c*_ij_ for the crystalline diamond and silicon.

As previously stated, in the presence of a pressure difference, the diamond diaphragm is compressed towards the fiber end-face, and the FP-cavity length is reduced by Δ*d* (see Figure 1a). The deflection of the circular sensor diaphragm, under a uniformly distributed pressure difference Δ*P* between the two faces, as schematically illustrated in Figure 3a, is given by [46]

(9) $$\Delta d\left(x\right)=\frac{12}{64}\left(\frac{1-\nu^{2}}{E}\right)\frac{1}{t^{3}}{\left(r^{2}-x^{2}\right)}^{2}\Delta P$$

where *t* and *r* are membrane thickness and radius, respectively, whereas *E* and *ν* are the Young’s modulus and Poisson’s ratio for diamond, respectively. As expected, at the diaphragm center (*x* = 0), Δ*d* assumes the maximum value. In Figure 3b, the results from the simulation are reported for three diamond membranes: 4, 6, and 8 µm thick, and with a diameter of 250 µm. On the vertical axis, the displacement values normalized to the applied pressure are reported. In this case, the values coincide with the sensor sensitivity. Mechanical deformations in the numerical simulations were calculated in the framework of the continuum model, minimizing the elastic energy from governing electromechanical equations based on Newton’s Second Law for a solid elastic crystal [47,48]. All of the models were implemented and solved using the TiberCAD simulator [49,50]. The linearly-independent elastic constants of the zincblende structure for the crystalline diamond and silicon are given in the table, reported as inset of Figure 3b [47]. As shown by the simulation results, the maximum deflection for *x* = 0 depends strongly on the thickness of the diaphragm, as well as its diameter. The thinner the diaphragm, the greater the sensitivity, but at the expense of a maximum pressure value that results in a λ/4 displacement. A sensor with a membrane diameter of 250 µm allows us to measure up to 0.6 MPa for a thickness of 4 µm, while with an 8 µm thick diaphragm, the limit is 4.3 MPa. Moreover, the former will have a sensitivity of 623 nm/MPa; the latter will have a sensitivity of 91 nm/MPa. 

**Figure 4:**
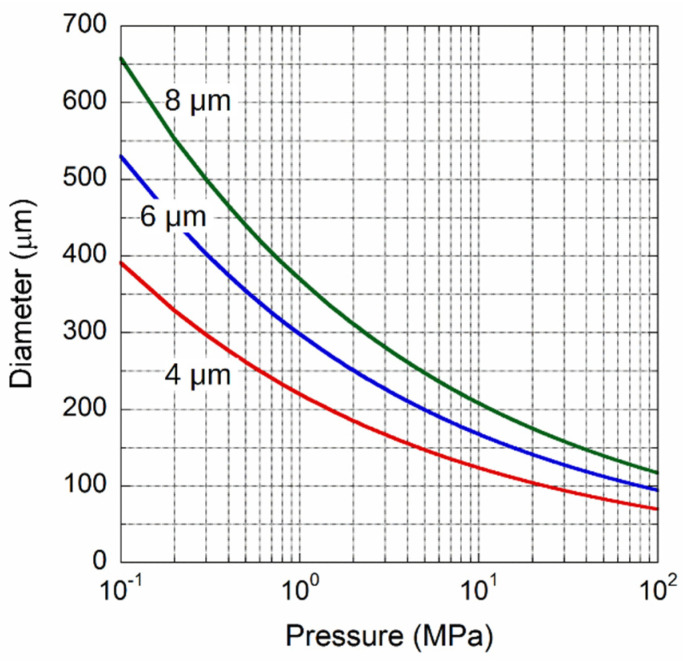
Calculated membrane diameter as a function of the pressure limit giving a displacement of 387.5 nm (λ/4) for film thicknesses of 4, 6, and 8 µm.

As mentioned before, the monotonicity of the photocurrent signal with the membrane deflection, and then with inlet pressure, occurs for a maximum membrane displacement of λ/4. Fixed to the thickness, the diameter of the diaphragm that will be displaced by λ/4 for a pressure limit-value can be calculated from Equation (9), and is reported in Figure 4. The data were calculated assuming λ = 1550 nm, and a Young’s modulus and Poisson ratio of 1100 GPa and 0.07 [51], respectively, for three diaphragm thicknesses. For example, for a sensor with a full-scale range of 1 MPa, a membrane with a thickness of 8 µm should have a diameter of about 370 µm, while for a thickness of 4 µm, a diameter of 220 µm is enough. As discussed in the following, a low thickness value should still ensure the continuity of the diamond film, and also mechanical properties comparable to those of crystalline diamond.

The diamond membranes were prepared using procedures described elsewhere [41]. A diamond film was grown by MPCVD on a 2 inch diameter, 350 µm thick, Si substrate. Uniform deposition was guaranteed by its being preliminarily seeded with nano-diamond (ND) powder (particle size of about 5 nm Daicel Corp., Osaka, Japan) by immersing the substrate for 10 min in an ultrasonic bath with a water-based suspension of ND particles. Then, polycrystalline diamond was deposited by means of a microwave plasma-assisted CVD equipment (ARDIS-100 system, 2.45 GHz, 5 kW, Optosystems Ltd., Troitsk, Russia) using a 6% CH_4_ in H_2_ gas mixture, with a total gas flow and pressure of 500 sccm and 55 Torr, respectively, maintaining the substrate temperature at 800 °C. At a growth rate of 1.2 µm/hour, after 5 h the film thickness was about 6 µm, assuring the full coverage of the substrate, as well as film continuity.

**Figure 5:**
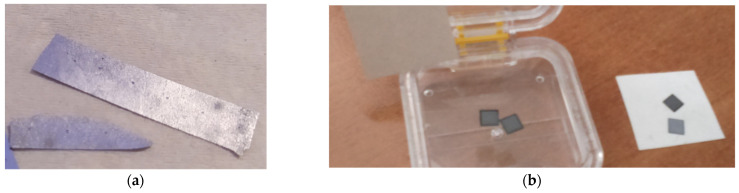
(**a**) An example of a thin diamond film grown on silicon. After the thinning of the silicon wafer up to a thickness of about 80 µm, substrate bending is clearly evidenced. (**b**) 5 mm × 5 mm diamond on 120 µm thick silicon plates after membrane fabrication.

After the diamond growth, the Si substrate back side was thinned to the thickness of 120 µm chosen as FP cavity length *d*_0_ using inductively-coupled plasma (ICP) etching (PLASMA TM5 system, NIITM, Moscow, Russia, operating at 13.56 MHz) with a SF_6_ + Ar gas mixture (with a flux of 6 and 4 L/min, respectively). The mentioned requirement of 43–62 µm to obtain an *I*_2_/*I*_1_ = 1 could not be satisfied: substrates that were too thin exhibited flatness problems. Indeed, Figure 5a shows the example of a substrate thinned down to 80 µm, in which the curvature of the sample is clearly visible. As a good compromise between light collection efficiency and sample robustness, a final thickness of 120 µm was chosen for the samples characterized in this work. In this case, the *I*_2_/*I*_1_ ratio would be 0.25 ± 0.05, and then a peak-to-peak/offset ratio of 8.5 ± 2.5. Although good performance is still expected in terms of the signal-to-noise ratio, it has to be observed that most of the offset signal the detector reveals comes from scattered incoherent light generated into the FP-cavity. Moreover, the unavoidable misalignment between the fiber and axis of the membrane may give rise to an inefficient collection of reflected light—i.e., a strong attenuation of *I*_2_—due to the non-negligible curvature of the diaphragm, as expressed by Equation (9). The experimental results discussed in Section 3 highlight this effect. It can be also observed that, in order to increase the intensity *I*_2_, and therefore the *I*_2_/*I*_1_ ratio, one could also resort to the deposition of a thin metal layer on the diamond nucleation side. It is noteworthy that the metal would be in the FP cavity, and not on the side exposed to the possibly-aggressive environment, and would therefore be protected by the diamond film itself.

After the mirror polishing of the silicon substrate and cleaning in deionized water, a 2 µm thick aluminum mask was deposited on the silicon by the electron beam physical vapor deposition technique (BAK 761, Evatec AG, Trübbach, Switzerland). A 0.1 µm thick Ti interlayer was preliminary deposited using the same equipment to promote a better adhesion of the aluminum mask to the silicon substrate. 

A 6 × 6 array of circular holes were created by the selective ICP etching of the silicon wafer after opening windows in the Ti/Al mask by ablation with a KrF excimer laser (CL-7100, 248 nm, pulse duration of 20 ns and repetition rate of 50 Hz, Optosystems Ltd., Troitsk, Russia). For this purpose, the same array of circular holes (with a diameter in the 3–8 mm range) of a tantalum mask were projected onto the Al/Ti surface with a 20× demagnification factor. The diameter of the holes obtained in the Ti/Al mask ranged between 150 and 400 μm, and were equispaced by 5 mm, i.e., resulting in about 5 mm × 5 mm as the dimension of each sensor. For the 6 µm thickness of the membranes, and the range of diameters between 150 and 400 µm, the sensors may have a full-scale pressure range of 0.3 to 15 MPa. 

As a final step, the metal layer was removed by means of chemical wet etching, and 5 mm × 5 mm plates were obtained by silicon slicing by means of cutting through both the Si substrate and PCD layer by the laboratory-built Nd:YAG laser system (λ = 1.06 µm), as shown in Figure 5b.

**Figure 6:**
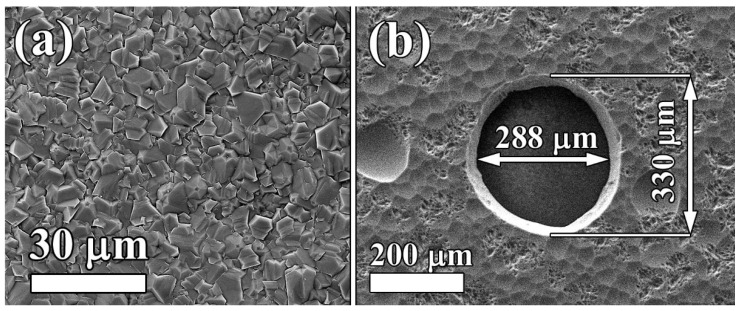
(**a**) SEM image of a 6 µm thick polycrystalline diamond on a silicon substrate. (**b**) SEM image of the nucleation side of the diamond membrane, viewed through a hole in the silicon substrate. The nominal value of the hole diameter was 300 µm.

**Figure 7:**
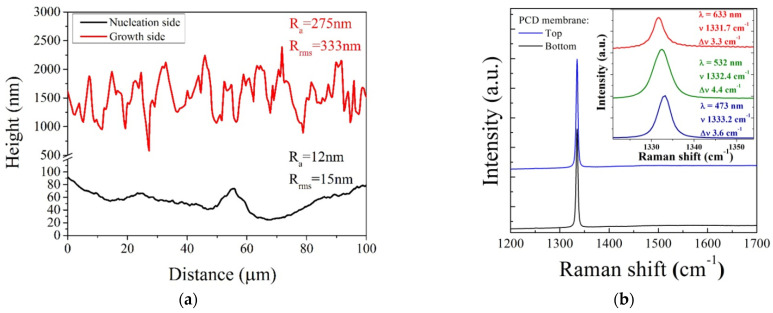
(**a**) Roughness of a polycrystalline diamond membrane surface at the growth and nucleation sides, as measured by optical profilometry. Note the difference in scale on the vertical axis for the two sides. (**b**) Raman spectra of the diamond membrane, taken at the 473 nm excitation wavelength on the top (growth) surface (blue spectrum) and fine-grained bottom surface (black spectrum). Inset: Raman spectra with a diamond peak at about 1332 cm^−1^ in the narrow range, taken at the 473, 532, and 633 nm wavelengths for growth surface. The peak position *ν* and width (FWHM) Δ*ν* are indicated.

In Figure 6, SEM images of a sample are reported. Randomly oriented well-faceted grains on the growth side are clearly revealed, with an average size of about 5 µm. The nucleation density (number of primary diamond particles per 1 cm^−2^) depends strongly on the seeding process. In our experiment, a nucleation density of the order of 10^9^ cm^−2^ was typical, which means the average distance was ~300 nm between neighbor seeds, and there was a similar size of extending grains on the substrate side after the growing seeds’ fusion. The diamond nucleation surface revealed by the silicon hole appears smooth, and its morphology is completely different from that of the growth side. In particular, an average and root mean square roughness *R_a_* and *R_rms_* of about 12 and 15 nm, respectively, were revealed on the nucleation surface, as reported in Figure 7a, as measured with a NewView5000 (ZYGO Corp., Middlefield, CT, USA) optical profilometer. Conversely, a roughness of around 0.3 µm was measured for the coarse-grain growth surface. The nearly flat surface on the side of the FP-cavity assures the low scattering of reflected light, and thus good light-collection by the fiber. 

Finally, micro-Raman spectroscopy (Labram HR-800, Horiba, France) was used to evaluate the diamond film quality on both sides of the membrane. A laser beam with a diameter of ~1.5 µm was focused on the center of an arbitrary selected diamond grain. The Raman spectrum taken on the growth side, taken at an excitation wavelength of 473 nm, revealed the sharp first-order diamond Raman peak at 1332.2 cm^−1^ with a full width at half maximum (FWHM) of 3.6 cm^−1^, as reported in Figure 7b, for the blue spectrum. The absence of non-diamond phase signatures in the spectrum evidences the high quality of the film. Quite a similar spectrum was obtained for the bottom (nucleation) side (see Figure 7b, black spectrum), which could be ascribed to the high transparency of the film both for excitation and the Raman wavelengths, so that the spectrum in each case is formed by the integration of the signal through all of the thickness. In addition, we measured Raman spectra taken at longer wavelengths of 532 and 633 nm that were similar to that obtained with 473 nm excitation. These multiwavelength spectra in a narrow range of 1320–1355 cm^−1^ are reported in the inset of Figure 7b in order to clearly show the width of each peak Δ*ν*. The full width at half maximum (FWHM) of the first-order peak varies between 3.3 and 4.4 cm^−1^, presumably due to the variation of the defect content and stress from grain to grain (the spectra were taken at different locations), rather than to the effect of the laser wavelength.

### 2.3. Sensor Assembly

**Figure 8:**
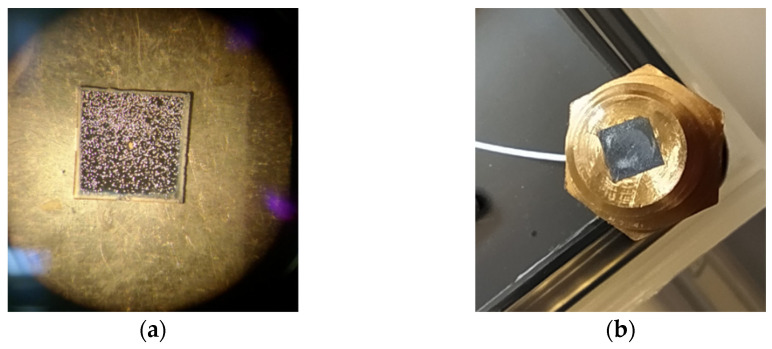
(**a**) Optical microscope image of a 5 mm × 5 mm diamond-on-silicon sample before membrane alignment and fixing on a brass holder. The bright spots are due to reflected light by the diamond microcrystals. In the center, the diamond membrane (~300 µm in diameter) is clearly visible, being completely transparent to white light. (**b**) A picture of a diamond membrane on silicon glued on the brass holder used for sensor characterization.

As indicated, the pressure sensor fabrication includes: (*i*) the growth of diamond film on silicon wafer and diaphragm realization; (*ii*) 5 mm × 5 mm sample realization by means of wafer slicing; (*iii*) optical fiber mounting on a housing system; and (*iv*) diamond diaphragm-SMF alignment and mounting. For the last two steps, a SMF with cladding and coating diameters of 125 and 245 µm, respectively (SMPF0215-FC, Thorlabs Inc., Newton, NJ, USA), terminated with a glass ferrule (1.8 mm in diameter), was inserted into a brass holder. The latter was mounted on a *x*–*y* micrometric stage, whereas the diamond-on-silicon membrane was maintained stable and gently pressed on the holder. Using the setup shown in Figure 2, the interference signal was detected during the *x*–*y* adjustment for alignment. When the ray beam impinged upon the membrane, the oscillation induced by the micrometric stage resulted in a variable signal observed on a digital oscilloscope (Agilent DSO-X 3024A, Keysight Technologies, Santa Rosa, CA, USA), whereas when the light beam was reflected by the silicon substrate, an almost-stable signal was observed. The maximum peak-to-peak amplitude was revealed when light impinged on the membrane center, i.e., the most sensitive and mobile part. Figure 8a shows an optical image of a sample mounted prior to SMF alignment. Having found the alignment, the 5 mm × 5 mm diamond-on-silicon sample was fixed with 1:1 mixable epoxy adhesive (Permabond^®^ ET515, Permabond Engineering Adhesives Ltd., Hampshire, UK) (see Figure 8b). In order to assure good adhesion, prior to the epoxy’s complete solidification, the holder with the glued sample was placed into the hydraulic table top test pump (P700.G, Sika, Kaufungen, Germany) at 1 bar for 24 h.

## 3. Results and Discussion

The characterization of the samples of different membrane diameters was conducted by means of the experimental setup illustrated in Figure 2, maintaining the power of light emitted by the fiber-coupled laser to 5 mW. Before the assembly and characterization of each sensor, the diameters of the diaphragms were evaluated by optical microscopy. For all of the values, an error of about ±10 µm has to be considered.

**Figure 9:**
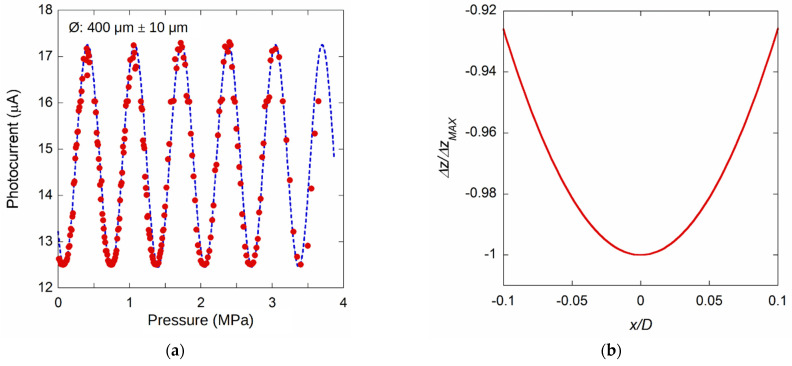
(**a**) Photocurrent signal acquired for a membrane of about 400 µm diameter, for pressures up to 3.6 MPa. The dotted line represents the best fit of the experimental data according to Equation (2). (**b**) A simulation of the displacement per unit of pressure normalized to the maximum, as a function of the *x* position normalized to the membrane diameter *D*.

Figure 9a reports a preliminary result in the 0–3.6 MPa pressure range for a sample with a diamond-membrane diameter of (400 ± 10) µm. The data shows the expected sinusoidal behavior, as indicated by Equation (2). The dotted line represents the best-fit result according to the mentioned equation. A membrane displacement of 775 nm (i.e., λ/2) occurs for a pressure change of 658 kPa, i.e., a sensitivity of around 1.2 nm/kPa. Taking into account the error on the diameter estimation, an *E*/(1−*ν*^2^) factor of (1180 ± 120) GPa is found by Equation (9), which is in good agreement with the values reported for diamond [51]. The mean photocurrent value is equal to 14.9 µA, whereas the peak-to-peak amplitude is 4.8 µA. Then, from Equation (2), evaluating the relative amplitude for *I*_1_ and *I*_2_, their *I*_2_/*I*_1_ ratio is of the order of 0.011, well below the 0.07–0.12 value expected for a 120 µm length of the FP cavity and reflectance values for diamond and SMF, as discussed in Section 2.1. It cannot be excluded that much of the loss of light intensity *I*_2_ must also be attributed to a slight misalignment between the fiber and the membrane axis. Indeed, subjected to a certain pressure *p*, it is not negligible the curvature of the diamond-membrane. Figure 9b highlights diaphragm curvature on the central position, showing the simulation results of the membrane displacement per unit of pressure, normalized to the maximum displacement, as a function of the *x* position, normalized to the membrane diameter, *D*.

**Figure 10:**
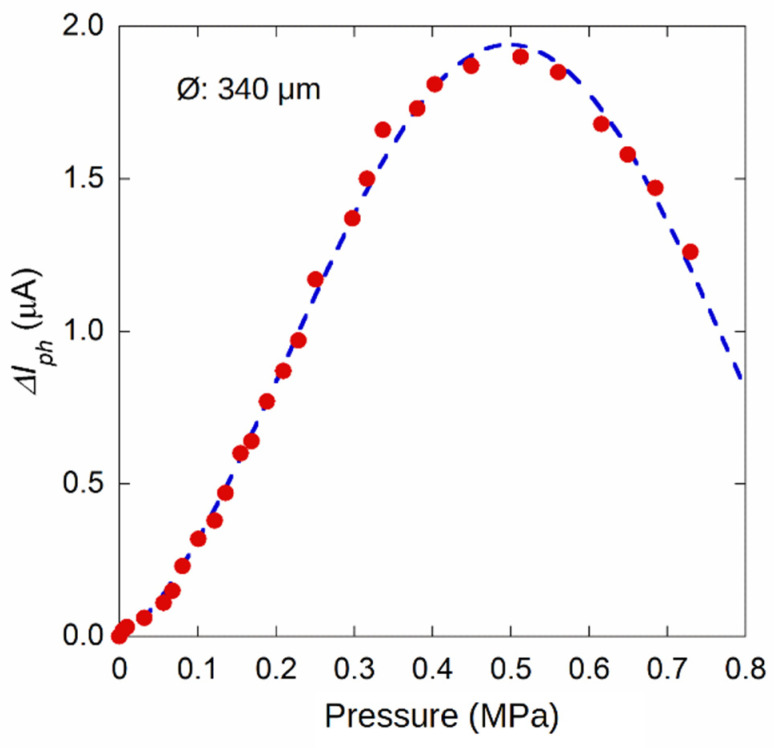
Photocurrent amplitude change with respect to the value acquired at *p* = 0 as a function of inlet pressure for a membrane sensor with a diameter around 350 µm.

Figure 10 displays the result obtained for another sensor with a membrane diameter of (350 ± 10) µm. In order to highlight the amplitude of the interference signal, the data reported in the figure refer to photocurrent signal acquired by subtracting the value recorded at *p* = 0. For such a sample, a monotonic behavior is well observed up to 0.5 MPa. Unlike the sample in Figure 9a, this sensor generates an increasing signal even at low pressures, indicating its possible use between 0 and 5 bar. In this case, the photocurrent signal can be acquired by dedicated readout electronics, in order to convert the current signal into the corresponding pressure value.

As aforementioned, due to the sinusoidal behavior of the output signal, a membrane pressure sensor coupled to the optoelectronic system used in this work needs to be employed in a more limited range, in which the signal displays a monotonic behavior. We conducted a systematic investigation on several samples, with membranes of different diameters, in order to select those that meet the requirement of generating an increasing or decreasing photocurrent signal in a relatively wide range of the inlet pressures. The selected elements were named M#, with membrane diameters in the 130–410 µm range, and 6 µm in thickness. Table 1 summarizes the main features of the sensors show in terms of pressure range and signal amplitude. In addition, by membrane diameter, the evaluated *E*/(1 − *ν*^2^) values were also reported.

**Figure 11:**
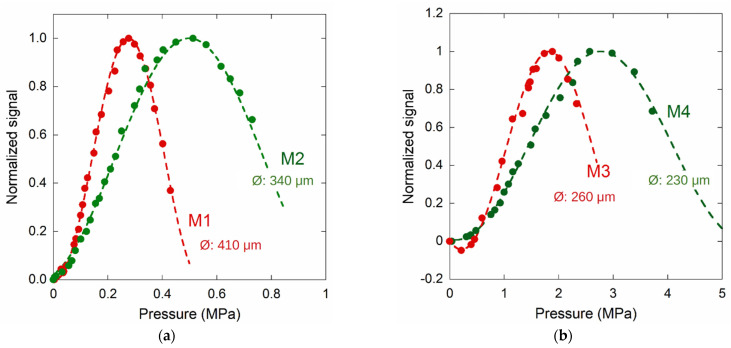
Normalized photocurrent signal induced by light wave interference for different diamond membranes (see text). Experimental results for M1 and M2, in (**a**), and for M3 and M4, in (**b**), membranes. See Table 1 for details.

**Figure 12:**
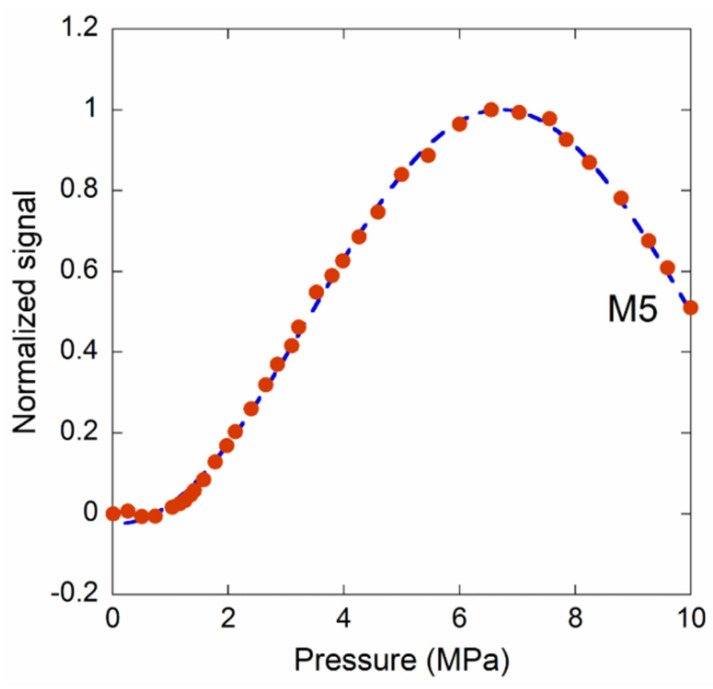
Normalized photocurrent signal induced by light wave interference for a sensor with a diamond membrane diameter of (190 ± 10) µm.

Figure 11 shows the obtained results for samples M1, 410 µm; M2, 340 µm; M3, 260 µm; and M4, 230 µm. In order to compare the sensor behaviors, each curve was normalized to its maximum. It is worthwhile to note that the M1 and M2 sensor outputs are monotonic from 0 Pa to 0.28 MPa, and from 0 Pa to 0.50 MPa, respectively. In order to extend the pressure range, M3 and M4—with a lower diaphragm diameter—display good results in the 0.2–1.9 MPa and 0–3 MPa ranges, respectively. Sample M3 displays the behavior found for the other samples (e.g., see Figure 9a): the signal is not monotonic at low pressure. Therefore, this sensor should be used to detect pressures greater than 0.2 MPa (2 bar). In order to overcome this matter, the position of the fiber relative to the membrane surface should be adjusted to nullify the phase shift φ_0_ at 0 Pa (see Equation (2)). The method to employ such a solution is under investigation. Finally, in Figure 12, the data of a pressure sensor with a diaphragm diameter of about 200 µm are reported. A monotonic signal was detected from tenths of an MPa up to about 7 MPa.

**Figure 13:**
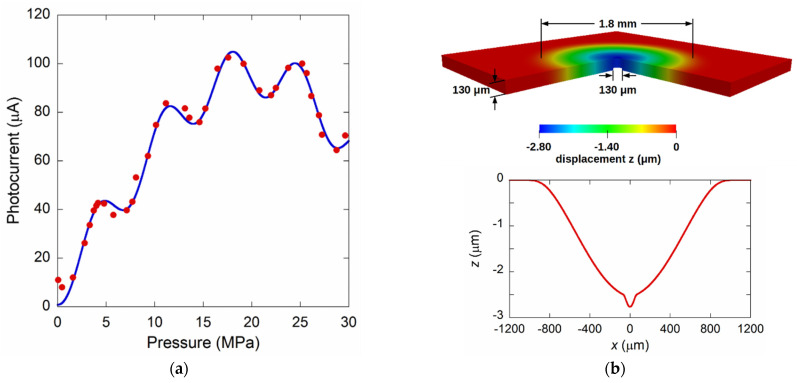
(**a**) Photocurrent signal acquired for a membrane sensor with a diameter of 130 µm. The continuous blue line represents best fit of data obtained considering the superposition of both the silicon and diamond displacement, according to Equation (9). (**b**) Numerical simulation of the structure in which the silicon substrate was considered unbounded on a circle of 1.8 mm diameter.

A completely different behavior was observed for the sample of which the data are shown in Figure 13. For the diameter of only 130 µm, we expected to be able to extend the measurement range up to about 20 MPa. The photocurrent signal is tentatively attributed to both the deflection of the diamond membrane and that of the silicon substrate. The blue line in Figure 13a represents the best fit of the experimental data using Equation (9) by linearly combining the contributions due to both the diamond and silicon. The best fit result was obtained assuming a silicon substrate of a thickness of 130 µm, in fairly good agreement to the value after the etching process, a Young’s modulus of 200 GPa, and a Poisson factor of 0.22 [52]. It is worthwhile to note that the experimental data are well approximated by the simulation, assuming for the substrate a diameter of 1.7 mm, which is almost coincident with that of the hole drilled in the brass-holder for glass-ferrule terminated fiber insertion, thus indicating the presence of a gap between the end of the fiber and the silicon substrate. Conversely, this phenomenon was not observed in other samples subjected to high pressure due to the insertion of the fiber-termination up to touch the silicon substrate. In addition, numerical simulations performed using the TiberCAD simulator for a pressure of 18 MPa confirmed the results, as reported in Figure 13b. The elastic constants of the crystalline structure for diamond and silicon are those reported as the inset of Figure 3b. The simulation clearly highlights a silicon displacement of about 2.5 µm and an added 0.3 µm displacement at *x* = 0 due to the diamond membrane. The results are in good agreement with those which were experimentally observed.

## 4. Conclusions

Diamond, for its particular mechanical characteristics, is a material particularly suitable for the realization of pressure sensors operating in aggressive environments, which are a requirement for various applications ranging from biomedical to automotive and industrial fields. The design, the realization, and the characterization of pressure sensors based on thin polycrystalline diamond membranes were reported. Numerical simulations were used for both the membrane’s design and the experimental results’ evaluation. The pressure-sensing element is represented by a thin diamond membrane used as a moving reflector in a 120 µm long FP-cavity. A 6 µm thick diamond film was deposited on a 2″ silicon substrate by means of the MPCVD technique. The selective etching of silicon allowed the definition of the diamond diaphragm’s diameter in the 130–410 µm range. Then, a single deposition-etching process allowed the fabrication of the different diamond-on-silicon membranes. Several 5 mm × 5 mm diamond membranes on silicon samples were obtained by substrate slicing by means of a Nd:YAG laser system. Each sample was mounted on a holder, and an SMF was aligned to the membrane. An optoelectronic system was used to evaluate the displacement of the diamond membrane as a function of the pressure difference applied to it. The assembled diamond-based pressure sensors were characterized in a wide pressure range, from a few bars to hundreds of bars, depending on the diaphragm diameter. The samples displayed non-negligible offset signals, and also good modulation depths due to the interference of light waves propagating into the FP-cavity, and directly related to the inlet pressure. Due to the relatively high peak-to-peak amplitude, the signal can be easily acquired and processed by a dedicated current-to-digital electronic system. Due to the optical fiber coupling, the system was found to be immune to EMIs. Furthermore, the proposed structure has only the diamond exposed to the environment of which the pressure is to be measured. Due to the physical characteristics and chemical inertness of diamond, such a sensor could then be used in hostile environments. In this context, a high-pressure chamber will be designed and realized in order to evaluate the performance of the sensors in high temperature regimes, or in the presence of aggressive agents.

## Figures and Tables

**Table 1:** Main characteristics of the selected sensors.

Sample	Diameter (µm)	P_MIN_ (kPa)	P_MAX_ (kPa)	I_ph_ @ *p* = 0 (µA)	Amplitude (µA)	*E*/(1 − *ν*^2^) (GPa)	Figures
M0	400	80	330	14.9	2.4	1180 ± 120	Figure 9a
M1	410	0	280	26	2.1	1070 ± 100	Figure 11a
M2	340	0	500	24.2	1.9	1030 ± 120	Figure 10, Figure 11a
M3	260	230	1900	17	2.9	1040 ± 160	Figure 11b
M4	230	0	2800	22	2.1	1050 ± 180	Figure 11b
M5	190	300	6800	19.4	2.4	1230 ± 260	Figure 12

## Data Availability

We did not report any data.
